# Antibiofilm Activity of Extract and a Compound Isolated from *Triumfetta welwitschii* against *Pseudomonas aeruginosa*

**DOI:** 10.1155/2021/9946183

**Published:** 2021-06-14

**Authors:** Molly Mombeshora, Godloves Fru Chi, Stanley Mukanganyama

**Affiliations:** ^1^Department of Biochemistry and Biotechnology, University of Zimbabwe, P. O. Box MP 167, Mt Pleasant, Harare, Zimbabwe; ^2^Department of Organic Chemistry, University of Yaoundé 1, Yaoundé, Cameroon

## Abstract

*Triumfetta welwitschii* has been used as a traditional medicine in Africa. It is documented as a rich source of phytochemicals with antibacterial activities. To further explore the antibacterial potential of these phytochemical components, the phytochemical profile of the dichloromethane: methanol leaf extract from *T*. *welwitschii* was investigated using ultra-performance liquid chromatography-tandem mass spectrometry (UPLC-MS/MS). Compounds were isolated from the extract using column chromatography and thin-layer chromatography. Compound B1 was isolated from the fraction eluted by 90 hexane:10 ethyl acetate using column chromatography. The antibacterial activity of B1 against *Pseudomonas aeruginosa* was evaluated *in vitro* using the broth microdilution method and the iodonitrotetrazolium (INT) colorimetric assay. The antibiofilm activities of the extract and B1 against *P*. *aeruginosa* were determined by quantifying the biofilms using crystal violet. The effect of the extract and B1 on capsular polysaccharide and extracellular DNA content of biofilm formed by *P*. *aeruginosa* was determined using phenol-sulphuric acid and propidium iodide, respectively. A total of 28 peaks were detected and identified using UPLC-MS/MS. The three most abundant phytochemicals identified were catechin, umbelliferone, and a luteolin derivative. B1 showed antibacterial activity against *P*. *aeruginosa* with a minimum inhibitory concentration (MIC) and minimum bactericidal concentration (MBC) value of 25 *μ*g/ml. Only 38% and 6% of the biofilms were formed in the presence of the extract and B1, respectively. The extract and B1 reduced the capsular polysaccharide content in biofilms formed in *P*. *aeruginosa* by 40% and 65%, respectively. The extract and B1 significantly reduced the extracellular DNA content of biofilms by 29% and 72%, respectively. The results of this study provide evidence of the antibacterial and antibiofilm activities of B1 and leaf extracts from *T*. *welwitschii*. Future work should identify the chemical structure of B1 using nuclear magnetic resonance and mass spectrometry.

## 1. Introduction

Antimicrobial agents play a vital role in reducing the global burden of infectious diseases. However, the emergence of resistant strains of pathogenic bacteria has become a major public health threat. The evolution of bacterial strains has rendered treatment protocols obsolete and highlights a paucity of antimicrobial agents that are effective against resistant bacterial strains [1]. The rapid global spread of resistant bacterial isolates necessitates the discovery of novel antimicrobial agents that control infections. Intracellular or biofilm-producing antibiotic-resistant bacteria are thought to be more virulent than other strains [2], which could be supported by the multicellular nature of biofilm bacterial communities. Extracellular polymeric substances (EPSs) are major constituents of biofilms. The EPSs enhance a biofilm community's ability to forage for both water and nutrients from the environment. Interestingly, this forage mechanism of EPS is not deterred in adverse environments, a phenomenon that enables biofilm-producing bacteria to persist in atypical conditions.

The invasion of implants like mechanical heart valves and catheters by biofilms has significant clinical impacts on patient outcomes [4]. The leading causes of nosocomial infections in humans have been associated with biofilm-forming pathogens, including [5] species of *Enterococcus faecalis*, *Staphylococcus aureus*, *Klebsiella pneumoniae*, *Acinetobacter baumannii*, *Pseudomonas aeruginosa*, and *Enterobacter* spp. These pathogens can “evade” the effects of antimicrobial treatment due to the acquisition of resistance genes and the formation of biofilms facilitated by EPSs. Targeting of the EPSs could disrupt biofilm physiology because a reduction in EPSs diminishes the hydrated barrier between cells and their external environment.

In addition to extracellular proteins and exopolysaccharides, extracellular DNA (eDNA) is an abundant constituent of the extracellular matrix of the biofilms formed by a wide range of bacteria. Ionic interactions of eDNA with Pel, a protein that sustains cell-to-cell interactions within biofilms, have been shown to be an essential component of antibiotic resistance [6]. The formation of biofilms by *P*. *aeruginosa* is a progressive process characterised by a high population of multiplicity. The formation of microcolonies is an initial step in this process. During the initial stages, eDNA is widely spread along the surface of the substrate and enables well-organised movement throughout the channel complex by sustaining articulate cell alignments, thus avoiding movement congestion and ensuring a proficient supply of cells to the migrating front [7]. If any step in the formation of the multicellular structure of the biofilm is disrupted, the effectiveness of antibiotics and the host defences might be improved, leading to better treatment outcomes [8]. The present study was designed to determine if B1 and a DCM: methanol extract from *Triumfetta welwitschii* had effects on the structure, capsular polysaccharide, and eDNA production of biofilms in *P*. *aeruginosa*.

*T*. *welwitschii* is of the Tilicea family and has been widely used in ethnomedicine because of its antimicrobial properties [9, 10]. Several biological activities have been reported in *T*. *welwitschii* extracts. These include antibacterial, antipyretic, and antimycobacterial activities [11–13]. *T*. *welwitschii* roots have been used to treat diarrhoea [9]. Moyo and Mukanganyama [14] have described the antiproliferative activity of root extracts from *T*. *welwitschii* against Jurkat cells. Detailed knowledge about the phytochemical profile and antibiofilm activity of the plant remains limited. The study also explored the phytochemical profile of the dichloromethane (DCM): methanol leaf extract from *T*. *welwitschii* by UPLC-MS/MS. Antibacterial activity of compound B1, which was isolated from *T. welwitschii* DCM: methanol extract, was evaluated against *P*. *aeruginosa*.

Several chromatographic methods may be utilised to profile phytochemicals in natural products. High-performance liquid chromatography (HPLC) is the preferred method to identify phytochemicals in natural products [15]. The coupling of HPLC with mass spectrometry (MS) and tandem MS (MS/MS) is effective in identifying phytochemicals at low concentrations when analysing complex samples [16]. However, a major drawback of this technique is the long analysis time, which ranges from 50 to 80 mins. Ultra-performance liquid chromatography (UPLC) is an improvement of the LC technique. UPLC employs chromatographic principles to separate compounds by utilising columns packed with smaller particles 1.7 *μ*m in size and/or columns with higher flow rates [17]. This technique leads to a shorter analysis time, higher peak efficiency, and higher resolution. Therefore, UPLC offers new possibilities of improving the analytical methods for complex samples that would otherwise require high resolution and long analysis times.

## 2. Materials and Methods

### 2.1. Chemicals Used in Assays

Chemicals used in the study included ciprofloxacin, dimethyl sulphoxide (DMSO), iodonitrotetrazolium (INT), and crystal violet were purchased from Sigma-Aldrich (Darmstadt, Germany). Tryptic soy broth (TSB) and tryptic soy agar (TSA) (22091) were also purchased from Sigma-Aldrich (Darmstadt, Germany).

### 2.2. Plant Material and Extraction

Leaves of *T*. *welwitschii* were collected from the centenary (16.8°S, 31.1167°E, and 1156 m above sea level). The plant's identity was authenticated by a botanist and a voucher specimen was deposited under the reference number C16 E7. The leaves were dried under shade for 14 days and then powdered to yield a sample with a mass of 2350 g. The powder was macerated with a mixture of DCM: methanol (1 : 1 *v*/*v*) for 48 hrs at room temperature. The extract was then concentrated under an RII rotary evaporator (BUCHI, LabortechnikAG, Switzerland) and then dried under a stream of air to create a residue (147 g) that constituted the crude extract.

### 2.3. UPLC-MS/MS Analysis of the DCM: Methanol Extract from *T. welwitschii*

Phytochemical fingerprinting of the extracts was performed as described by Thomford et al. [18]. A Waters ACQUITY UPLC system (Waters Corporation, Milford, MA, USA) with an ACQUITY BEH C18 column (2.1 mm × 100 mm, 1.7 *μ*m particle size) that inluded a binary pump, vacuum degasser, autosampler, column oven, and Micromass Xevo tandem quadrupole mass spectrometric detector (QTOF Xevo G2; Waters Micromass, Manchester, UK) equipped with an electrospray ionisation (ESI) probe was used. Gradient elution was performed at a flow rate of 0.1 ml/min throughout at injection volumes of 10 *μ*l. Gradient parameters were adjusted by systematically changing the percentage of organic modifier at initial conditions, the isocratic hold period at initial conditions, and/or gradient steepness. Electrospray mass spectra data were recorded in a negative ionisation mode for a mass ranging from 100 *m*/*z* to 1500 *m*/*z* at a collision energy of 50 V. MassLynx v.4.1 software (Waters) was used to determine the accurate mass and composition for the precursor ions and fragment ions. Fragmentation data, retention times, and data from relevant literature [19–21] were used to identify compounds that may be found in the extracts; standard compounds and the KNApSAcK species-metabolite relationship database were used to assign metabolites.

### 2.4. Isolation of Compound(s) Using Column Chromatography

An extract portion weighing 120 g was mixed with silica gel (480 g) column chromatography (70–230 mesh, Merck), eluted with varying mixtures of hexane, ethyl acetate, and methanol stepwise gradients to obtain 450 fractions (250 ml each). The column had a diameter of 6 cm and a length of 42 cm. Thin-layer chromatography (TLC) of the fractions collected was run on aluminium silica gel 60 F254 (MERCK) plates and viewed under an A425/G Allen Ultraviolet light lamp (P. W. Allen & Co., London, UK) at 254 nm and 365 nm. The spots representing separated compounds were further detected with 10% sulphuric acid. The TLC plate was exposed to heat at 100°C until carbon-charred spots became visible. The fractions of *T. welwitschii,* which were initially purified through silica gel column chromatography, did not show distinctively separated spots on TLC plates. The fractions were washed with ethyl acetate for further purification. The washed fractions were subjected to TLC. TLC studies of the residue collected on filter paper and the filtrate itself were completed using silica gel aluminium plates. Chloroform was used as the mobile phase for the separation of compounds. Visualisation of TLC plates was carried out under an ultraviolet lamp at 254 nm and 365 nm and further detection by 10% sulphuric acid. Compound B1 was isolated.

### 2.5. Bacterial Strain Used in Assays

The American Type Culture Collection *P*. *aeruginosa* (ATCC 27853) was acquired from the Microbiological Section in the Department of Biological Sciences at the University of Botswana (Gaborone, Botswana). Bacteria were kept as glycerol stocks at −35°C.

### 2.6. Determination of the Antibacterial Activity of B1 against *P. aeruginosa*

The antibacterial activity of B1 isolated from the DCM: methanol leaf extract of *T*. *welwitschii* against *P. aeruginosa* was determined using the broth microdilution assay [22]. Cells were grown overnight at 37°C in TSB. A 0.5 McFarland standard was used to calculate the volume of inoculum required to produce a value of 2 × 10^6^ c.f.u/ml used in minimum inhibitory concentration (MIC) determination. Concentrations of 0 to 100 *μ*g/ml of B1 were used in the microdilution assay. Ciprofloxacin was used as a standard antibacterial agent for the assays. A Genios Pro 96-multiwell microplate reader (Tecan Group, Ltd., Mannedorf, Switzerland) was used to obtain a preincubation reading before the plates were incubated at 37°C for 24 hrs. A postincubation reading was taken after the incubation period. The growth of cells was quantified using the difference in optical density at 590 nm between the preincubation reading and the postincubation reading. A visual confirmatory test using the INT assay [23] was used to observe and spectrometrically quantify metabolically active cells at 590 nm using a Genios Pro microplate reader. The colour changes observed were red for viable cells and yellow for nonviable cells. The MIC was determined as the lowest concentration that showed no growth. The minimum bactericidal concentration (MBC) was determined using samples from the MIC microtiter plate. A loopful of inoculum was collected from the well with the concentration before the well that had been read as the MIC. The samples were plated onto TSA, and bacterial growth was observed after incubating the plates for 24 hrs at 37°C. The lowest concentration of B1 showing no bacterial growth was recorded as the MBC.

### 2.7. The Effects of the Extract and B1 on Biofilms

#### 2.7.1. The Effect of the Extract and B1 on Biofilm Formation

*P*. *aeruginosa* was grown at 37°C for 24 hrs in TSB supplemented with 2% glucose. The optical density (OD) was measured at 600 nm using a spectrophotometer (Model S2100, Unico Science, New Jersey, USA) and appropriate dilutions were made in TSB + 2% glucose to obtain an optical density of 2 × 10^6^ cells/ml using the 0.5 McFarland standard. The assay was completed in a sterile 24-well polystyrene plate (Corning® Costar® TC, Merck). In brief, 1000 *μ*l of *P*. *aeruginosa* cells were inoculated and cultured with or without 1000 *μ*l of 100 *μ*g/ml extract or B1, without shaking at 37°C. Bacterial cells without extract or B1 were used as positive controls. TSB +2% glucose was used as a negative control. TSB +2% glucose + extract (not inoculated) and TSB +2% glucose + B1 (not inoculated) were included as additional negative controls. After 72 hrs of incubation, nonadherent cells were removed by washing each sample three times in sterile phosphate buffer solution (PBS). Plates were dried by inverting them on absorbent paper for 15 mins. Samples were fixed at 60°C for 1 hr and the biofilms were stained with 1000 *μ*l of a solution of 0.1% crystal violet in water, a method modified from O'Toole [24]. Plates were incubated at room temperature for 15 mins. Samples were washed thrice with PBS. The quantitative analysis of biofilm production was completed by adding 1250 *μ*l of 95% ethanol to destain the samples. The quantity of biofilms was determined at 590 nm using a microplate reader (Tecan Austria GmbH, Grödig, Austria). The percentage of biofilm inhibition was determined by the following formula:

(1) $$\begin{array}{c} \text{Percentage biofilm growth}\left(\%\right)=\frac{\text{OD sample}}{\text{OD control}}\times 100. \end{array}$$

#### 2.7.2. Microscopic Analyses of Biofilms

Microscopic analysis of the effects of the extract and B1 on biofilm structure was carried out by staining the biofilms using a crystal violet stain. An overnight culture was standardised to 2 × 10^6^ c.f.u/ml using a 0.5 McFarland standard. Test extract or compounds (100 *μ*g/ml) and cells were dispensed in a ratio of 10 ml: 10 ml in Petri plates containing a sterile glass slide. Petri plates were incubated at 37°C for 72 hrs. A plate with TSB only was included as a control for sterility. After the incubation period, 2.7 ml of 1.5% SDS in PBS (v/v) was added to the plates. The plates were further incubated at 37°C for a further 30 mins. The slides were removed from the Petri plates aseptically and washed with PBS. The bacterial biofilms were fixed to the slides using 2% sodium acetate and stained with 0.1% crystal violet. The slides were washed and air-dried. The biofilm was visualised under a light microscope (Nikon, Tokyo, Japan) at 40× magnification.

#### 2.7.3. Evaluation of the Biofilm Disruption Potential of the Extract and B1

To determine the effect of the extract and B1 on biofilms developed over 72 hrs, plates were prepared in the same manner as the inhibition assay, but without the addition of test samples, and incubated for 72 hrs. Cells were washed and test samples (100 *μ*g/ml) were dispensed into the wells. The 24-well plate was then incubated for a further 24 hrs and biofilms were quantified as before.

#### 2.7.4. Determination of the Effects of the Extract and B1 on the Quantity of Capsular Polysaccharides

Polysaccharide extraction was done using a process modified from Wu et al. [25] with modifications. In brief, a culture of *P*. *aeruginosa* cells grown for 16 hrs was standardised using a 0.5 McFarland standard to 1 × 10^9^ c.f.u/ml. The cells were grown in 50 ml centrifuge tubes containing 100 *μ*g/ml of extract or B1. A final concentration of 0.25 *μ*g/ml of ciprofloxacin was used as the standard antibacterial drug. Each tube had 5 ml of cells and 5 ml of extract, ciprofloxacin, or B1. Unexposed cells and media without cells were included as the positive and negative controls, respectively. The cell cultures were incubated in a water bath at 37°C for 4 hrs without shaking. After the incubation period, cells were separated by centrifugation at 4 000 rpm for 15 mins and washed three times with PBS. The cells were suspended in 450 *μ*l of deionised water and an equal volume of saturated phenol. The mixture was incubated for 20 mins in a water bath at 65°C. A volume of 300 *μ*l from each tube was transferred in triplicate to 1.5 ml microtubes (Eppendorf, Sigma-Aldrich, Darmstadt, Germany). A volume of 150 *μ*l chloroform was added and the mixtures were mixed by intense vortexing. The cell suspension was centrifuged and the supernatant was collected. The supernatant was prepared for phenol-sulphuric acid assay [26]. In brief, 50 *μ*l of each supernatant was distributed into wells on a microtiter plate. Next, 150 *μ*l of concentrated sulphuric acid (98%) and 5% phenol were added to each well. Mannose was used as the standard sugar, and a standard curve was plotted. The standard curve was prepared by placing 50 *μ*l of each mannose concentration of 20, 40, 60, 80, and 100 *μ*g/ml into wells on a microtiter plate. Concentrated sulphuric acid (98%) and 5% phenol were added to the standards in the same manner as for the sample supernatant. The microtiter plate was placed in a static water bath at 90°C for 5 mins. The plate was dried and the absorbance was read at 492 nm using a Stat fax 2 100 microplate reader (Awareness Technologies, Inc., Westport, USA).

#### 2.7.5. Determination of the Effects of the Extract and B1 on the Quantity of eDNA

The eDNA was extracted using a procedure modified from work by Chiba et al. [27]. The quantity of extracted eDNA was determined fluorimetrically using the propidium iodide stain [28]. In brief, an overnight culture of cells was grown at 37°C at 120 rpm. The overnight culture was standardised to 2 × 10^6^ c.f.u/ml using a 0.5 McFarland standard. The cells were centrifuged at 3500 rpm for 15 mins and the supernatant was discarded. The remaining pellet was washed using PBS. A volume of 1 ml of the resulting solution was pipetted into a 24-well plate and 1 ml of the extract or B1 (at a final concentration of 100 *μ*g/ml) was added to respective wells. A positive control comprising cells without treatment and a negative control comprising cells treated with ciprofloxacin, the reference drug, were included. TSB was included as a sterility control. The plate was incubated at 37°C for an additional 72 hrs in a humidified atmosphere in a shaking incubator. The nonadherent contents of Plate 1 were transferred to a new 24-well plate (Plate 2) using a 1 ml pipette. The first plate was washed using PBS by running 2 ml of the buffer. The plate was inverted over a paper towel for 15 mins to drain any excess liquid. Each well of Plate 1 was stained using 900 *μ*l PBS + 2 mM MgCl_2_ and 5 *μ*l propidium iodide stock solution. Plate 2 was stained with 5 *μ*l/well of propidium iodide. The mixture was kept in the dark for 10 mins at room temperature. A volume of 200 *μ*l of the test samples was transferred to a 96-well plate and fluorescence was measured at excitation and emission wavelengths of 544 and 612 nm, respectively, using an *f*_max_ spectrofluorometer (Molecular Devices, Sunnyvale, USA).

### 2.8. Statistical Analyses

The data from the results obtained in this study were analysed using GraphPad Prism for Windows (GraphPad Software Inc., San Diego, California, USA) version 8.0.1. A one-way analysis of variance (ANOVA) test and Dunnett's multiple comparison test were used to determine the level of significance; all treated samples were compared to the control. Values with *P* < 0.05 were considered statistically significant.

## 3. Results

### 3.1. Chemical Composition of a Leaf Extract from *T. welwitschii*

**Figure 1:**
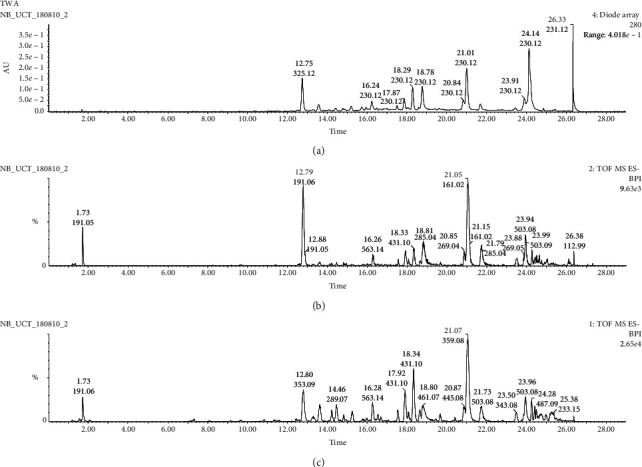
UPLC-MS/MS chromatogram of crude DCM: methanol extracts of *T. welwitschii*. The retention time was in minutes. Major peaks of 161, 191, and 359 *m*/*z* showed a relative abundance of 100%. Detection and analyses of the extracts were performed using a Waters ACQUITY UPLC system.

A UPLC-MS/MS chromatogram of the DCM: methanol leaf extract from *T. welwitschii* showing a total of 28 peaks with varying relative abundances was depicted in Figure 1. Three dominant peaks of 161, 191, and 359 *m/z* were detected.

A total of 28 physiologically active components were identified from the extract by UPLC-MS/MS analysis. Results indicated that catechin, umbelliferone, and a derivative of luteolin were the major compounds in the leaf extract from *T*. *welwitschii*. The identified components are summarised in Table 1 according to their retention times.

**Figure 2:**
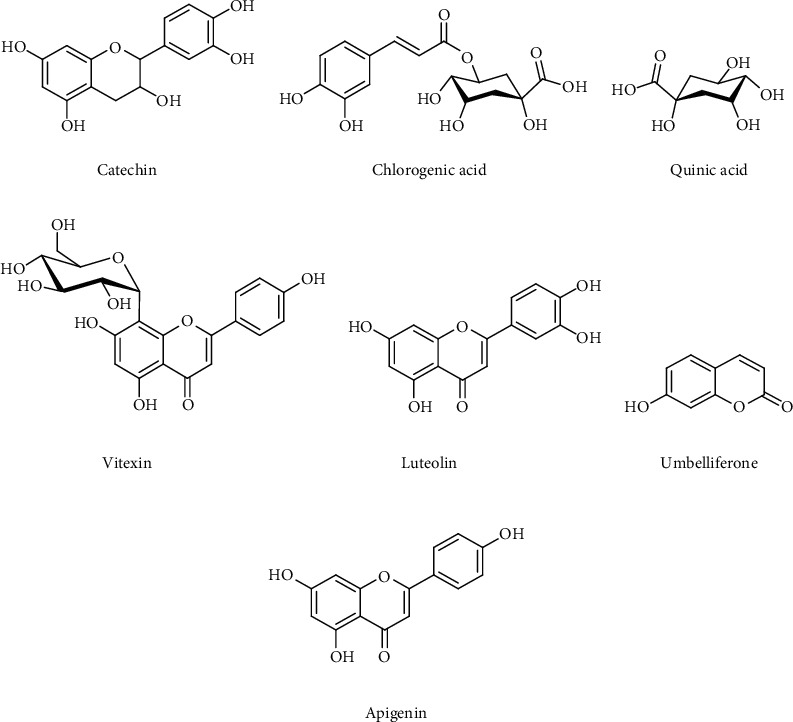
Chemical structures of some of the compounds identified from the DCM: methanol leaf extract of *T. welwitschii* using UPLC-MS/MS analysis.

The proposed compounds mainly belonged to the flavone, flavanol, phenol, coumarin, and cyclic polyol classes. Chemical structures of some compounds identified from the DCM: methanol extract using UPLC-MS/MS analyses are shown in Figure 2.

### 3.2. Isolation of B1 from the Leaf Extracts of *T. welwitschii*

A total of 450 fractions were obtained from column chromatography of the DCM: methanol leaf extract from *T*. *welwitschii*. Fractions with similar TLC profiles were combined to yield 27 pools. The 27 pools had a mixture of many impure compounds as indicated by multiple spots on TLC plates. Pooled fractions were washed with solvents of higher polarity and spotted on TLC plates to detect the number of compounds present. TLC plates were developed using chloroform as the mobile phase. Plates were viewed under 254 nm and 365 nm (UV). B1 showed a distinct single spot, while the rest of the fractions showed some tailing along with spots. The presence of a single spot from B1 implied that a potential pure compound had been isolated. MB1, FA27, A29, and FA29 had single spots with some tailing. A27 and FA1 had two and three spots, respectively.

### 3.3. Antibacterial Activity of B1

**Figure 3:**
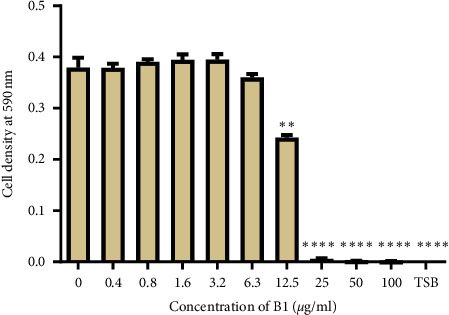
The effects of B1 on the growth of *P*. *aeruginosa*. TSB: tryptic soy broth. Values are expressed as mean OD at 590 nm wavelength ± the standard deviation (*n* = 4). The difference between the control and other sample treatments was tested at a 95% confidence interval. The *asterisks* indicate a significant difference from the positive control with ^*∗∗*^ indicating *P* < 0.01 and ^*∗∗∗∗*^ indicating *P* < 0.0001. B1 showed antibacterial activity from a concentration of 6.3 *μ*g/ml. The MIC of B1 against *P*. *aeruginosa* was 25 *μ*g/ml.

The effect of B1 on the growth of *P*. *aeruginosa* was determined using the broth microdilution method. B1 showed a concentration-dependent inhibition on the growth of *P*. *aeruginosa* starting from 6.3 *μ*g/ml up to 25 *μ*g/ml. Total inhibition of bacterial growth was observed at concentrations ≥25 *μ*g/ml, as shown in Figure 3. The MBC for B1 was found to be 25 *μ*g/ml.

### 3.4. The Effects of the Extract and B1 on Biofilm Formation

**Figure 4:**
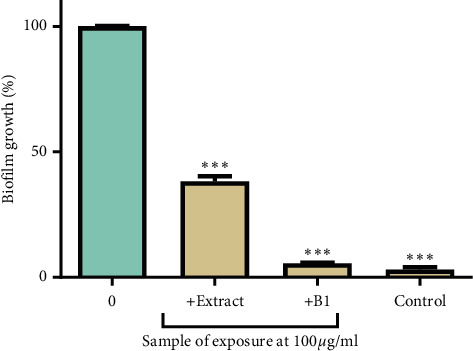
The effects of the extract and B1 on biofilm formation in *P. aeruginosa*. The control contained tryptic soy broth without cells. The error bars indicate the standard deviation from the mean (*n* = 4). The asterisks (^*∗*^) indicate statistically significant differences compared to the positive control (unexposed *P. aeruginosa*), which represented cells without any extract, where ^*∗∗∗*^ denotes *P* < 0.0001. At 100 *μ*g/ml, the extract and B1 resulted in only 38% and 6% formation of biofilm in microplates by *P*. *aeruginosa,* respectively.

The formation of biofilm by *P*. *aeruginosa* in the presence and absence of the extract or B1 at 100 *μ*g/ml is presented in Figure 4. There was a significant difference in the biofilm formed by bacteria exposed to all three test samples when compared to the biofilm formed in bacteria not exposed to the test samples.

**Figure 5:**
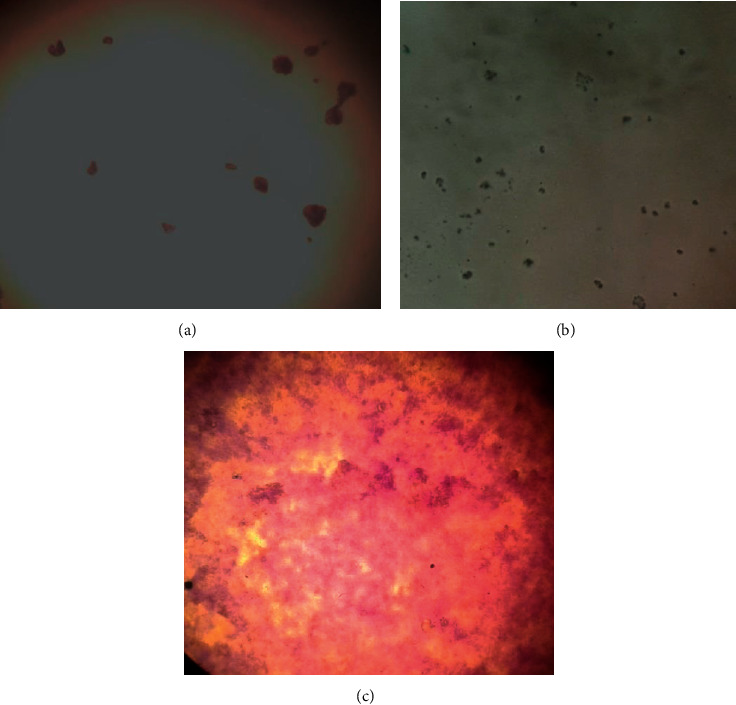
Images of the effects of the extract and B1 on biofilm structure of *P*. *aeruginosa* viewed under a Nikon light microscope at 40x magnification. (a) Cells treated with the extract had a dispersed biofilm structure. (b) Cells treated with B1 had no biofilm structure. (c) The untreated cells showed a compact biofilm structure.

The structure of biofilm after 72-hour exposure to test samples was analysed under light microscopy. Biofilms exposed to the extract or B1 showed disrupted structure, while unexposed biofilm retained a compact structure. Images of the effects of the extract and B1 on biofilm structure obtained using a microscope are included in Figure 5.

### 3.5. Disruption of Mature Biofilms

**Figure 6:**
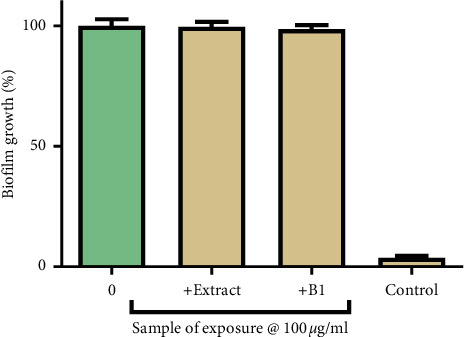
The effects of the extract and B1 on the growth of mature biofilms by *P*. *aeruginosa*. Control contained tryptic soy broth media without cells. The error bars indicate the standard deviation from the mean (*n* = 4). The control is unexposed *P. aeruginosa*. The extract and B1 had no significant effect on the growth of mature biofilm.

The effect of the extract and B1 on preformed biofilms in 24-well plates was determined using the crystal violet assay. Both the extract and B1 were unable to disrupt the preformed biofilm, as shown in Figure 6.

### 3.6. The Effects of the Extract and B1 on the eDNA Content of Biofilms

**Figure 7:**
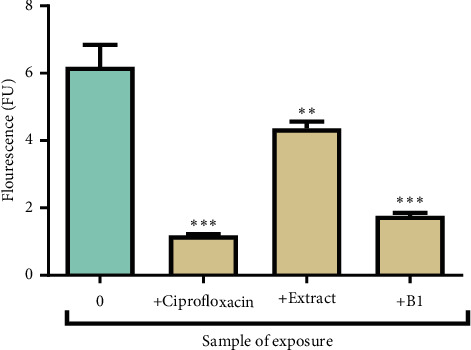
The effects of the extract, B1, and ciprofloxacin on the production of eDNA by *P*. *aeruginosa.* Values indicate mean ± standard deviation for *n* = 4. The asterisks indicate a significant difference from the control (unexposed *P. aeruginosa*) with ^*∗∗*^ indicating *P* < 0.01 and ^*∗∗∗*^ indicating *P* < 0.001. The extract and B1 reduced the content of eDNA produced by 29% and 72%, respectively.

The extract, B1, and ciprofloxacin significantly reduced the production of eDNA by *P*. *aeruginosa* compared to the untreated biofilms. The untreated biofilms produced the highest quantity of eDNA compared to treated cells. The effects of the extract and B1 on the production of eDNA in biofilm are presented in Figure 7.

### 3.7. The Effects of the Extract and B1 on the Content of Extracellular Polysaccharide (EPS) in Biofilms

**Figure 8:**
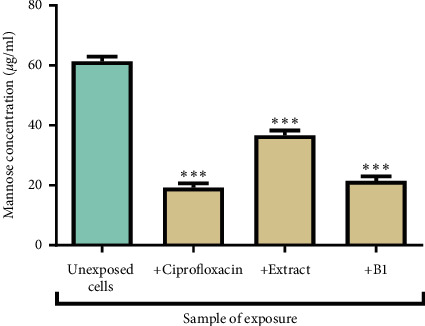
The effect of ciprofloxacin, the extract, and B1 on capsular polysaccharide content in biofilms formed by *P*. *aeruginosa*. The error bars indicate the standard deviation from the mean (*n* = 3). The asterisks (^*∗∗∗*^) indicate statistically significant differences from the positive control (cells without any extract) at a significance level of *P* < 0.0001. The content of capsular polysaccharides was reduced by 40% and 65% after treatment with the extract and B1, respectively.

The amount of capsular polysaccharide remaining after biofilm treated under different conditions was interpolated from the standard curve of mannose. Biofilms treated with ciprofloxacin, the extract, or B1 had significantly lower sugar content compared to the untreated biofilms. Biofilms exposed to ciprofloxacin had the lowest amount of sugar compared to all other treatments. The effects of the extract and B1 on the content of EPS in *P*. *aeruginosa* biofilms are presented in Figure 8.

## 4. Discussion

### 4.1. Identification of Compounds in *T. welwitschii* Using UPLC-MS/MS

Antibacterial activities of extracts from *T. welwitschii* have been previously reported [11] and the DCM: methanol extract has been found to be the most potent. UPLC-MS analysis of the DCM: methanol extract from *T*. *welwitschii* led to the identification of 28 phytochemicals. The majority of compounds identified belonged to the flavonoid class. Several flavonoids are known to exhibit antioxidant, antibacterial, antifungal, and antiviral activities [29]. The three most abundant compounds identified from the extract were catechin, umbelliferone, and a luteolin derivative. The flavonoid catechin has many reported positive effects on human health, such as anticancer, antiobesity, antidiabetic, anticardiovascular, antimicrobial, hepatoprotective, and neurological benefits [30]. Irreversible damage to the microbial cytoplasmic membrane has been reported as the antimicrobial mechanism of catechins [31]. Luteolin is another bioactive flavonoid. The antimicrobial activity of *Achillea tenuifolia* against *Staphylococcus aureus*, *Bacillus subtilis*, and *Enterococcus faecalis* has been attributed to two derivatives of luteolin [32]. The plant-derived phenolic coumarin umbelliferone has been found to possess antifungal and antibacterial activities. Mazimba [33] has reported bioactivities of umbelliferone against inflammation and tumour cells. The biological activities observed for the extracts of *T*. *welwitschii* may be attributed to these bioactive compounds. A summary of the pharmacological roles of the other compounds detected and identified in the DCM: methanol leaf extract from *T*. *welwitschii* is included in Table 2.

### 4.2. Isolation of B1 from the Leaf Extracts of *T. welwitschii*

Chromatography can be used for separation or quantitative analysis [43]. Separation should be achieved within a suitable time period. In this work, TLC was used to determine the number of compounds in each fraction obtained from column chromatography, followed by washing with ethyl acetate. Some molecules of the samples were colourless; thus, fluorescence and 10% sulphuric acid were used to create a detectable coloured product to observe spots on the chromatogram. The formation of visible colour was observed under UV light. Fraction B1 showed a pure band on the TLC plate, while fractions MB1, FA27, A29, and FA29 showed one band and some tailing. Fractions B1 may contain a pure compound [44], while fractions FA27, A29, and FA29 may require further purification. The tailing observed for the three fractions may be an indication that they contain alkaloids [45]. Fractions FA1 and FA27 had more than two spots, indicating that they may contain more than one compound in them.

### 4.3. Antibacterial Activity of B1

A large number of medicinal plants have been documented as valuable sources of natural antimicrobial compounds that may offer effective alternatives in the treatment of problematic bacterial infections. Antibacterial activity of the DCM: methanol, ethanolic, and acetone extracts from *T*. *welwitschii* was reported in a previous study [11]. The most potent extract was reported as the DCM: methanol extract, with an MIC of 100 *μ*g/ml and an MBC of greater than 100 *μ*g/ml. Extracts prepared from the DCM: methanol solvents were used to isolate and purify the active compounds that may be responsible for bioactivity. B1 was isolated from the extract using column chromatography and TLC. The antibacterial activity of B1, the compound isolated from *T*. *welwitschii,* was determined in the current study. According to the present results, B1 had an antibacterial activity with a MIC and MBC of 25 *μ*g/ml. The isolated compound showed greater antibacterial activity compared to the crude extract, suggesting that purification aided in the separation of the compounds interfering with the activity of the extract [46].

### 4.4. The Effects of the Extract and B1 on the Formation of Biofilms

Antibiofilm activities of compounds from natural products may play an essential role in bacterial infections associated with medical devices. Pathogens can resist antimicrobials more when they exist in biofilms as infection can persist on different biotic and abiotic surfaces [47]. Factors that cause resistance in biofilms include the presence of an extracellular polymeric matrix, which causes the strong attachment of microbes to surfaces and low antibiotic penetration or increased activity of efflux pumps that expel antimicrobial agents from cells [48]. The extract and B1 inhibited biofilm formation (Figure 4). The extract and B1 may have interfered with any of these factors. The extract and B1 may have also interfered with cell-to-cell communication strategies (quorum sensing) of the bacteria, thereby reducing biofilm formation [49].

The effects of the extract and B1 on *P*. *aeruginosa* biofilm structure were analysed using a light microscope. Cells treated with the extract showed a greater reduction in the biofilm thickness compared to the untreated culture. The extract evidently inhibited the production of virulence factors and biofilm formation [50] in *P*. *aeruginosa*. Cells treated with B1 and ciprofloxacin had no biofilm structure nor any viable cells. The antibiofilm effects of natural products may be attributed to the inhibition of the formation of the polymer matrix, consequently hindering the quorum sensing network and the development of biofilm [51]. This effect may have left the cells exposed and more susceptible to the antimicrobial effects of B1 and ciprofloxacin.

### 4.5. The Effects of the Extracts and B1 on Mature Biofilms

The extract and B1 did not have any disruptive effect on mature (72 hr) biofilms of *P*. *aeruginosa* was observed. Once biofilms have been established, they tend to exhibit more resistance to external agents such as antibiotics, detergents, or biocides than their planktonic cells [52]. Therefore, disruption of mature biofilms tends to require higher doses of disrupting agents than those needed to destroy planktonic cells [53]. Slow or incomplete penetration of the antimicrobials into the established biofilm population [54] or an altered biochemical microenvironment within the biofilm increases the difficulty of disrupting mature biofilms [8].

### 4.6. The Effects of the Extract and Compound on Extracellular Polysaccharide Content

The phenol-sulphuric acid assay was used to determine the carbohydrate content in the cells exposed to different conditions. In the presence of strong acids and heat, carbohydrates undergo a series of reactions that lead to the formation of furan derivatives such as furanaldehyde and hydroxymethyl furaldehyde [55]. The role of the EPS in the pathogenesis of biofilms has been studied in many organisms in an attempt to evade the widespread infectious effects of biofilms. The adhesion and resistance-related allow them to synthesize and secrete exopolysaccharides [3]. The extract and B1 induced a decrease in the capsular polysaccharide content. Ciprofloxacin reduces bacterial biomass by inhibiting DNA gyrase [56]; reduced biomass may be related to the reduction of the bacterial density that consequently reduces polysaccharide production. The reduction of the exopolysaccharides after exposure to the extract and B1 suggests that the production of exopolysaccharides by surviving cells might also be impaired [57]. The underlying mechanism for such reduction of exopolysaccharide production after exposure to antimicrobials remains unknown, and investigating the regulatory cascade leading to exopolysaccharide production would be a promising research direction. Otani et al. [58] have suggested that the *β*-lactam ceftazidime may weaken the polysaccharide matrix synthesis of *P*. *aeruginosa* through a reduction in the production of Pel and Psl exopolysaccharides. Reducing the amount of Pel amount might have been a mechanism of polysaccharide content reduction by the extract and B1.

### 4.7. The Effects of the Extract and B1 on eDNA Production

Biofilm has eDNA as a structural component that binds biofilm during formation and shields it from antimicrobials, giving resistance mechanisms to *P. aeruginosa*. The release of eDNA plays an important part in the mechanism of action of antibiofilm agents [59]. The extract and B1 significantly reduced eDNA produced by the biofilm of *P*. *aeruginosa*. This finding supports results from a study by [60], which found a significant reduction in the amount of eDNA in the biofilm matrix of biofilm treated with hamamelitannin. In the formation of biofilm by *P*. *aeruginosa*, eDNA is an adhesion agent that facilitates cell-to-cell attachment stabilising the biofilm and augments resistance against degrading agents [61]. Therefore, an agent that can decrease the amount of eDNA may reduce the formation of biofilm.

## 5. Conclusion

Compounds detected and identified by UPLC-MS/MS analysis may be responsible for the biological activities of extracts from *T*. *welwitschii*. B1 may be a pure compound. Further work to identify its structure is to be carried out using nuclear magnetic resonance and mass spectrometry. B1 possesses antibacterial and antibiofilm activity against *P*. *aeruginosa*. The DCM: methanol extract and B1 significantly reduced the content of capsular polysaccharides in *P. aeruginosa* biofilm. The DCM: methanol extract and compound B1 from *T*. *welwitschii* inhibited the production of eDNA and may be one of the mechanisms of disrupting biofilm formation by *P*. *aeruginosa*. Thus, phytochemicals from *T*. *welwitschii* may serve as antibiofilm and antibacterial lead compounds for targeting infections due to *P*. *aeruginosa*.

## Figures and Tables

**Table 1:** The proposed identification of compounds corresponding to the chromatographic peaks in Figure 1 by UPLC-MS/MS.

Peak	Rt (min)	[M-H]^−^	Proposed formula	Proposed compounds
1	1.73	191.0546	C_7_H_11_O_6_	Quinic acid
2	1.73	191.0549	C_7_H_11_O_6_	Quinic acid
3	12.79	191.06	C_7_H_11_O_6_	Quinic acid
4	12.80	353.0874	C_16_H_17_O_9_	Chlorogenic acid
5	12.88	191.05	C_7_H_11_O_6_	Quinic acid
6	14.46	289.07	C_7_H_11_O_6_	Catechin
7	16.27	563.1394	C_20_H_30_O_7_	Apigenin 6-C-arabinoside 8-glucoside
8	16.26	563.1393	C_20_H_30_O_2_S_2_	Apigenin 6-C-arabinoside 8-glucoside
9	17.92	431.0961	C_21_H_9_O_10_	Vitexin
10	18.34	431.0978	C_21_H_9_O_10_	Vitexin
11	18.80	461.07	C_22_H_22_O_11_	Methylkaempferol-hexose
12	18.81	285.0397	C_15_H_10_O_6_	Luteolin
13	20.85	269.04	C_15_H_9_O_5_	Apigenin
14	20.87	445.08	C_21_H_18_O_11_	Apigenin-7-*O*-glycuronyl
15	21.05	161.0239	C_9_H_6_O_3_	Umbelliferone
16	20.87	445.08	C_21_H_18_O_11_	Apigenin-7-*O*-glycuronyl
17	21.08	359.08		Luteolin derivative
18	18.80	461.07	C_22_H_22_O_11_	Methylkaempferol-hexose
19	21.07	359.08		Luteolin derivative
20	21.15	161.02	C_9_H_6_O_3_	Umbelliferone
21	21.73	503.08	C_23_H_19_O_13_	6,8-Di-*C*-*β*-glucupyranosylapigenin (vicenin-2)
22	21.79	285.04	C_15_H_10_O_6_	Luteolin
23	23.50	343.08		5,4′-Dihydroxy-7,3′-dimethoxy8-methyl homoisoflavanone
24	23.96	269.0441	C_15_H_9_O_5_	Apigenin
25	23.96	503.0821	C_23_H_19_O_13_	6,8-Di-*C*-*β*-glucupyranosylapigenin (vicenin-2)
26	23.99	503.09	C_23_H_19_O_13_	6,8-Di-*C*-*β*-glucupyranosylapigenin (vicenin-2)
27	25.38	233.15		Malonyl- monocinnamoylquinic acid
28	26.38	112.99		Quinic acid derivative

**Table 2:** Summary of pharmacological roles of compounds identified in the DCM: methanol leaf extract from *T*. *welwitschii* using UPLC-MS/MS.

Proposed compounds	Pharmacological role	References
Quinic acid	Antibacterial activity	[34]
Chlorogenic acid	Antioxidant and anti-inflammatory activities	[35]
Apigenin 6-C-arabinoside 8-glucoside	Antioxidant and lipophilicity activities	[36]
Vitexin	Anti-inflammatory, antioxidant, cardioprotective, antcancer, and antidiabetic activities	[37]
Methylkaempferol-hexose	Antioxidant and anti-inflammatory activities	[35]
Apigenin	Antifungal activity and cytotoxicity on colon cancer cells	[38]
Apigenin-7-*O*- glucuronyl	Antifungal activity and cytotoxicity on colon cancer cells	[38]
6,8-Di-*C*-*β*-glucupyranosyl apigenin (vicenin-2)	Antioxidant, antiviral, anti-inflammatory, and hepatoprotective	[39]
5,4′-Dihydroxy-7,3′-dimethoxy8-methyl homoisoflavanone	Anti-inflammatory and antihyperglycemic activities	[40, 41]
Malonyl-monocinnamoylquinic acid	Antibacterial and antifungal activities	[42]

## Data Availability

The datasets generated and analysed during the current study are available from the corresponding author on reasonable request.
